# Effect of Fushengong Decoction on PTEN/PI3K/AKT/NF-κB Pathway in Rats With Chronic Renal Failure via Dual-Dimension Network Pharmacology Strategy

**DOI:** 10.3389/fphar.2022.807651

**Published:** 2022-03-15

**Authors:** Hongyu Luo, Munan Wang, Ke Xu, Qiyao Peng, Bo Zou, Shi Yin, Chao Yu, Lingyan Ren, Ping Li, Li Tang, Yongbo Peng, Xuekuan Huang

**Affiliations:** ^1^ Chongqing Key Laboratory of Traditional Chinese Medicine for Prevention and Cure of Metabolic Diseases, College of Traditional Chinese Medicine, Chongqing Medical University, Chongqing, China; ^2^ Chongqing Key Laboratory for Pharmaceutical Metabolism Research, The Key Laboratory of Biochemistry and Molecular Pharmacology, College of Pharmacy, Chongqing Medical University, Chongqing, China; ^3^ School of Safety Engineering, Chongqing University of Science and Technology, Chongqing, China; ^4^ Department of Anesthesiology, The First Affiliated Hospital, Chongqing Medical University, Chongqing, China; ^5^ Radiation Oncology Center, Chongqing University Cancer Hospital and Chongqing Cancer Institute and Chongqing Cancer Hospital, Chongqing, China

**Keywords:** Fushengong decoction, chronic renal failure, dual-dimension network pharmacology, UHPLC-MS/MS, PTEN/PI3K/AKT/NF-κB

## Abstract

**Overview:** The treatment of chronic renal failure (CRF) with traditional Chinese medicine has attracted much attention, but its mechanism is not clear. Network pharmacology is an effective strategy for exploring the interaction mechanisms between Chinese herbs and diseases, however, it still needs to be validated in cell and/or animal experiments due to its virtual screening characteristics. Herein, the anti-CRF mechanism of the Fushengong decoction (FSGD) was investigated using a dual**-**dimension network pharmacological strategy combined with *in vivo* experiment.

**Methods:** The traditional Chinese medicine systems pharmacology (TCMSP) database (https://tcmspw.com) and UHPLC-MS/MS technology were used to identify the effective compounds of FSGD in theory and practice, such as quercetin, formononetin, and pachymic acid. The putative targets of FSGD and CRF were obtained from the Swisstarget prediction platform and the Genecards database, respectively. The common target pathways between FSGD and CRF were got from the dual-dimension network pharmacology analysis, which integrated the cross-common targets from the TCMSP components-Swisstarget-Genecards-Venn platform analysis in theory, and the UHPLC-MS/MS identified effective ingredients-Swisstarget screening, such as TNF and PI3K/AKT. Furthermore, system molecular determinations were used to prove the dual-dimension network pharmacology study through CRF rat models, which were constructed using adenine and treated with FSGD for 4 weeks.

**Results:** A total of 121 and 9 effective compounds were obtained from the TCMSP database and UHPLC-MS/MS, respectively. After dual-dimension network pharmacology analysis, the possible mechanism of PTEN/PI3K/AKT/NF-κB pathway was found for FSGD in CRF. *In vivo* experiments indicated that FSGD can play a role in protecting renal function and reducing fibrosis by regulating the PTEN/PI3K/AKT/NF-κB pathway. These findings provide a reference for FSGD in CRF.

**Conclusion:** Based on the theoretical and practical dual-dimension network pharmacology analysis for FSGD in CRF, the possible molecular mechanism of PTEN/PI3K/AKT/NF-κB was successfully predicted, and these results were verified by *in vivo* experiments. In this study, the dual-dimension network pharmacology was used to interpret the key signal pathway for FSGD in CRF, which also proved to be a smart strategy for the study of effective substances and pharmacology in FSGD.

## 1 Introduction

Chronic renal failure (CRF) is a disease caused by many causes of progressive loss of renal function that eventually produce renal failure (Mehta et al., 2020). The prevalence rate in developed and developing countries is as high as 10.8–16.0% (Yuan et al., 2019). Recently, CRF has become a major public healthcare problem that cannot be ignored because of its critical condition, high morbidity, and mortality. It has been shown that the pathogenesis of CRF may be closely related to genetic factors, hemodynamic changes, inflammatory factors, and oxidative stress, etc (Ullah and Basile, 2019). The main treatment methods for CRF are kidney transplantation and hemodialysis, but the curative effect is often poor.

Traditional Chinese medicine (TCM) has received great attention for improving the quality of life and survival rate of patients with CRF (Wang et al., 2012). The Fushengong decoction (FSGD, Supplementary Table S1) was summarized by the master and professor of Chinese medicine, Ziguang Guo, who added and deducted some herbs in the traditional Chinese medicines (TCM) formula “Liuwei Dihuang Pill” with 60 years of clinical experience. Its functions are to tonify the kidney, promote blood circulation, and reduce turbidity to improve renal function and renal fibrosis (Yang et al., 2017). In our previous study, FSGD can significantly lowered the expression levels of α-SMA and TGF-β1 to reduce renal fibrosis (Wang et al., 2016; Tong et al., 2020). Although the clinical application of FSGD in CRF has been verified, the mechanism of FSGD in treating CRF remains unclear because of the complexity of its components and targets. Therefore, further mechanism study of FSGD in CRF is very valuable.

**FIGURE 1:**
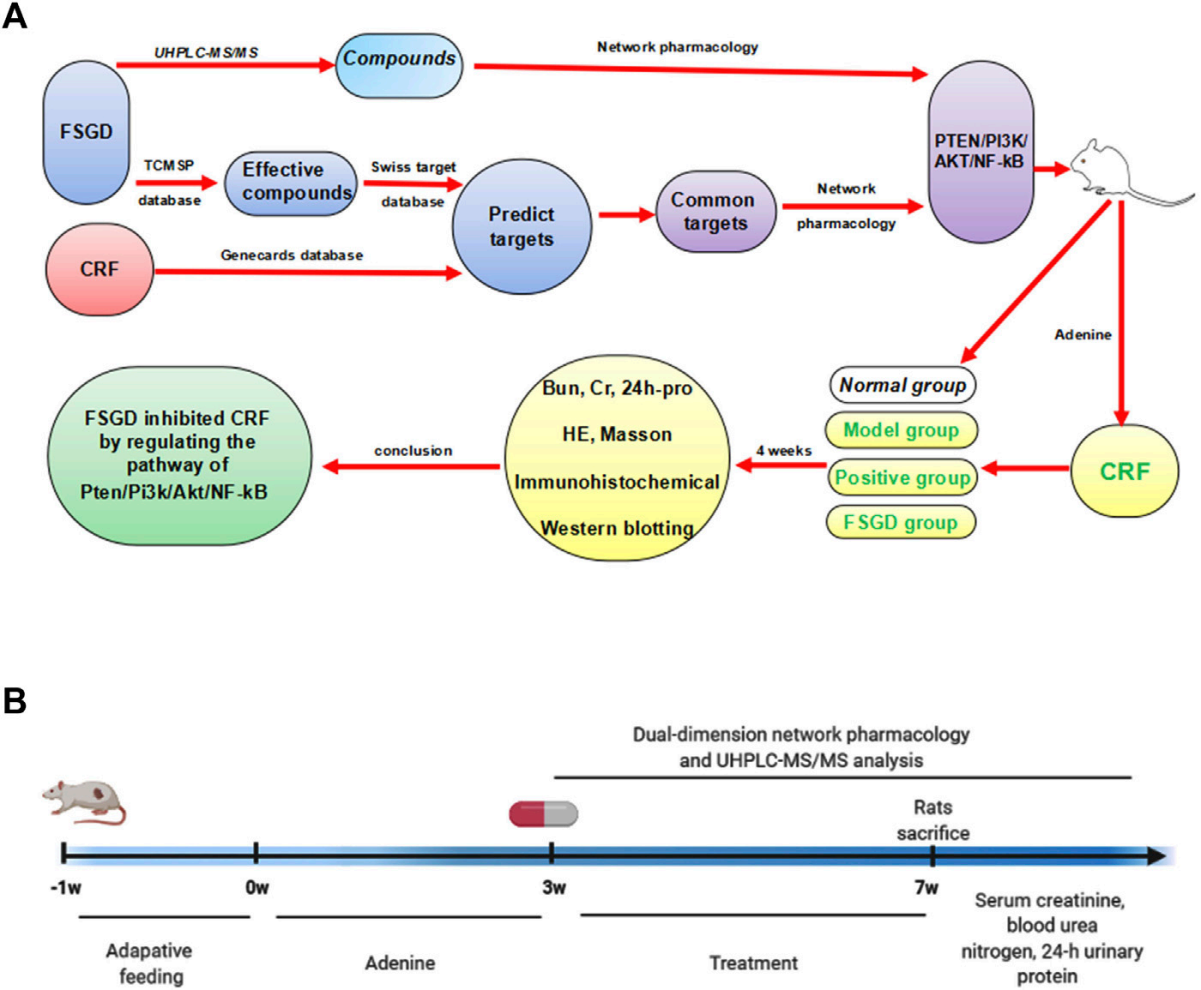
The study schematic illustration of the mechanism for FSGD in CRF. **(A)** Process of experiments. **(B)** Timeline of experiments.

Network pharmacology is an effective strategy for exploring the interaction mechanisms between chinese herbs and diseases. Because of the complexity of components and multi-targets in TCM, network pharmacology has the advantages of predicting the potential mechanism of prescription action in the treatment of diseases through compound-target-disease interaction network and bioinformatics analysis, which is consistent with the holistic perspective of TCM (Wang X et al., 2020). However, it still needs to be validated in cell and/or animal experiments due to its virtual screening characteristics. To explore the anti-fibrosis mechanisms of FSGD on CRF, dual-dimension network pharmacology of the effective components of the database and the compounds detected by UHPLC-MS/MS were used to predict candidate compounds and mechanisms of FSGD in the treatment of CRF. And *in vivo* experiment was conducted to further confirm the pathway. An overview of this study is showed in Figure 1 by BioRender (https://biorender.com/).

## 2 Materials and Methods

### 2.1 Materials

Niaoduqing granules (NDQ, Z20073256) were obtained from kangchen pharmaceutical Co., Ltd., and 0.5% adenine was purchased from Jiangsu Nantong trofi feed company (Jiangsu China); serum creatinine (Cr), serum urea nitrogen (BUN), and urine protein detection kits were obtained from Nanjing Jiancheng Bioengineering Institute (Nanjing, China). Antibodies against PTEN (48756), PI3K (48848), p-PI3K (11508), AKT (48888), p-AKT (11054) and NF-κB (48498) were obtained from Signalway antibody (United States).

### 2.2 Preparation of Fushengong Decoction

FSGD is composed of huangqi (*Astragalus mongholicus* Bunge), dihuang [*Rehmannia glutinosa* (Gaertn.) DC.], shanyao (*Dioscorea oppositifolia* L.), shanzhuyu (*Cornus officinalis* Siebold & Zucc.), cheqian (*Plantago asiatica* L.), niuxi (*Achyranthes bidentata* Blume), mudanpi (Paeonia × suffruticosa Andrews), zexie [Alisma plantago-aquatica subsp. orientale (Sam.) Sam.], fuling [Poria Cocos (Schw.) Wolf], cangzhu [*Atractylodes lancea* (Thunb.) DC.], duzhong (*Eucommia ulmoides* Oliv.), shuizhi (*Hirudo* Whitman) and huangbai (Phellodendron chinense C. K. Schneid.)*.* The herbal information and composition ratio are shown in Supplementary Table S1 *.* FSGD herbs were selected according to the “Chinese pharmacopoeia” 2020 edition. All herbs are purchased from the famous Tongjun Pavilion in China. These herbs were identified by professor Xuekuan Huang of Chongqing Medical University and preserved in the Chongqing Key Laboratory of Traditional Chinese Medicine for Prevention and Cure of Metabolic Diseases. Combined with the surface area conversion between rats and humans (6.3) during daily dosage of adults, the daily dose of rats was calculated to be 8 g/kg. In the last 7 years, low, medium, and high doses of FSGD was evaluated for the treatment of adenine-induced CRF in rats, respectively. Our previous experiments showed that 8 g/kg is the reasonable dose for the treatment of CRF (Wang et al., 2016; Tong et al., 2020). According to the effective therapy (8 g/kg) and the preparation of traditional decoction, FSGD was soaked for 30 min with purified water and boiled three times every 30 min. The boiling liquid was collected, filtered, concentrated to crude drug (1 g/ml), and stored at 4°C for use (Wang et al., 2016; Yang et al., 2017; Tong et al., 2020). Part of the FSGD decoction was stored at −80°C for further analysis.

### 2.3 UPLC-QTOF-MS Analysis

The samples of FSGD were thawed in ice water, vortexed for 30 s, centrifuged at 12,000 rpm at 4°C for 15 min. A 300 μl aliquot of sample was precisely transferred to an eppendorf tube. After the addition of 1,000 μl extracting solution (methanol: water = 4:1, *v/v*, including internal standard concentration is 10 μg/ml), all samples were vortexed for 30 s, sonicated for 5 min in an ice-water bath, incubated at −40°C for 1 h, and centrifuged at 12,000 rpm at 4°C for 15 min. A 500 μl of the supernatant was passed through a 0.22 μm filter membrane and then transferred to ultra-high-performance liquid chromatography tandem mass spectrometry (UHPLC-MS/MS) analysis.

LC-MS/MS analysis was performed on a Agilent ultra-high performance liquid chromatography 1290 UPLC system with a Waters UPLC BEH C18 column (1.7 μm × 2.1 mm × 100 mm). The column temperature was set at 55°C and the sample injection volume was set at 5 μl. The flow rate was set at 0.5 ml/min. The mobile phase consisted of 0.1% formic acid in water (A) and 0.1% formic acid in acetonitrile (B). The multi-step linear elution gradient program was as follows: 0–11 min (85–25% A), 11–12 min (25–2% A), 12–14 min (2–2% A), 14–14.1 min (2–85% A), 14.1–15 min (85–85% A), 15–16 min (85–85% A). An Q Exactive Focus mass spectrometer coupled with an Xcalibur software was employed to obtain the MS and MS/MS data with the IDA acquisition mode. During each acquisition cycle, the mass range was from 100 to 1,500, and the top three of every cycle were screened and the corresponding MS/MS data were further acquired. Sheath gas flow rate: 45 Arb, Aux gas flow rate: 15 Arb, Capillary temperature: 400°C, Full MS resolution: 70000, MS/MS resolution: 17500, Collision energy: 15/30/45 in NCE mode, Spray Voltage: 4.0 kV (positive) or −3.6 kV (negative). Mass spectra were imported raw using XCMS software. Materials identification of peaks containing MSMS data was performed using the secondary mass spectrometry database (Shanghai BIOTREE biotech Co., Ltd.) and the corresponding cleavage law matching method.

### 2.4 Animal Experiments

Eight-week-old male Sprague-Dawley rats (200 ± 20 g) were purchased from the Laboratory Animal Center of Chongqing Medical University and kept in a specific-pathogen-free level at the center (SCXK 2018-0003). The animal study was reviewed and approved by the Ethics Committee of Chongqing Medical University. After 1 week of adaptive feeding, all rats were randomly divided into four groups (*n* = 8), normal group, model group, NDQ group, and FSGD group. NDQ was used as the positive drug, which is widely used in clinical practice and is a commonly used drug for CRF (Zheng et al., 2017). The normal group was fed a regular diet, while the other group was fed a diet supplemented with adenine by gavage at a dose of 0.5% adenine to induce the CRF model for 3 weeks according to our previous method (Wang et al., 2016; Yang et al., 2017; Tong et al., 2020). After 3 weeks, CRF mice were randomly divided into three groups (*n* = 8). Rats in the normal and model groups were treated with physiological saline, FSGD (8 g/kg) and NDQ (5 g/kg), respectively, by gavage for 4 weeks. At the end of the experimental period, all rats were fasted for 12 h and sacrificed. Blood samples were then collected and centrifuged at 3500 rpm for 10 min, and the serum was collected and stored at −80°C until further analyses.

### 2.5 Network Pharmacology Analysis

#### 2.5.1 Identification of the Theoretical Active Compounds and Protein Targets of FSGD

FSGD effective compounds were collected from the traditional Chinese medicine systems pharmacology (TCMSP) database (http://lsp.nwsuaf.edu.cn) using the ADME filter method, and the main parameters included oral bioavailability (OB) and drug-likeness (DL). The effective compounds were defined based on the values of OB of ≥30%, DL of ≥0.18 and half-life (HL) ≥4 (Su et al., 2007). The compounds with CRF treatment were identified as potential compounds in FSGD via text mining. The CAS number of each compound was uploaded to the pubchem platform (https://pubchem.ncbi.nlm.nih.gov/) to obtain its 2D structure, and the swiss target prediction platform (http://www.swisstargetprediction.ch/) was used to identify compound-related targets, zero probability, and repeated targets were removed. Moreover, the human gene symbol corresponding to the protein target name was standardized using the uniProt database (https://www.uniprot.org/).

#### 2.5.2 CRF Related Target Prediction

Target genes associated with CRF were acquired from genecards (https://www.Genecards.org/). The targets common in FSGD and CRF were identified by the venn platform and used for further analysis (http://bioinformatics.psb.ugent.be/webtools/Venn/).

#### 2.5.3 GO and KEGG Pathway Enrichment Analysis

To obtain the potential signaling pathway associated with FSGD in the treatment of CRF, KEGG analysis was conducted by david (https://david.ncifcrf.gov), and GO analysis was conducted using metascape (http://metascape.org/).

### 2.6 Detection of Metabolic and Biochemical Indicators

Serum Cr, BUN and 24 h urine protein level were assessed according to the manufacturer’s instructions. Then, 24 h urinary protein quantification was calculated based on urine volume and urinary protein concentration.

### 2.7 Histological and Immunohistochemical Assays

According to the manufacturer’s instructions, kidney tissues were fixed in 4% paraformaldehyde for 24 h, dehydrated, embedded in paraffin, and cut into 4 μm sections for hematoxylin-eosin (HE) imaging, Masson staining, and immunohistochemical (IHC) experiments. All the sections were analyzed by microscopy (BX53, Olympus Corporation, Japan). The cumulative optical density was collected and calculated using Image-Pro Plus software (Media Cybernetics, United States).

#### 2.7.1 Hematoxylin-Eosin Staining

The paraffin sections were dewaxed to water, dip-stained with hematoxylin staining solution for 4 min, flushed with distilled water, dehydrated with gradient alcohol, and then stained with eosin staining solution for 5 min, dehydrated and sealed.

#### 2.7.2 Masson Staining

The sections were soaked overnight with 2.5% potassium dichromate mordant, stained with ferric hematoxylin staining for 2 min, rinsed with distilled water, differentiated with 1% hydrochloric acid alcohol for 30 s, rinsed with distilled water, transferred into lichun red acidic fuchsin dye for 6 min. And the lichun red sections were rinsed with distilled water, soaked in 1% phosphomolybdic acid for 50 s, rinsed with distilled water, dyed with 2.5% aniline blue for 20 s, rinsed and differentiated with 1% glacial acetic acid. Xylene transparent, gum sealed sheet. After sealing, the slides were examined and analyzed under microscope.

#### 2.7.3 Immunohistochemical Experiments

The sections were incubated with citrate antigen retrieval solution for 20 min at 95°C, and primary antibody [PTEN (1:100), PI3K (1:100), AKT (1:100), and p-AKT (1:100)] and secondary antibody was incubated with these sections for overnight and 50 min, respectively. The cumulative optical density was collected and calculated with Image-Pro Plus software (Media Cybernetics, United States).

### 2.8 Western Blotting Analysis

Protein expression levels were detected using antibodies against PTEN (1:5,000), PI3K (1:2,000), p-PI3K (1:500), AKT (1:5,000), p-AKT (1:1,000) and NF-κB (1:2,000). GAPDH (1:5,000) was used as a control. Target proteins were separated by sodium dodecyl sulfate polyacrylamide gel electrophoresis and transferred to polyvinylidene fluoride membranes. These membranes with target proteins were then blocked with 5% skim milk for 1.5 h and incubated with primary and secondary antibodies. Enhanced chemiluminescence reagents were added to the membrane and target proteins were visualized with a chemiluminescence imaging system (Odyssey Fc, LI-COR Biosciences, United States).

### 2.9 Statistical Analysis

Statistical analyses were performed on the SPSS 20.0 software (IBM, United States), and using one-way analysis of variance (ANOVA) followed by a least significant difference (LSD) test or Dunnett’s T3 test. All experiments in this study were independently repeated thrice to ensure reliable results. Values are expressed as mean ± standard deviation.

## 3 Results

### 3.1 Information of the Theoretical Active Compounds and Related Targets of FSGD

A total of 121 FSGD compounds were retrieved from the database of TCMSP. OB ≥ 30% and DL ≥ 0.18 were recognized as compound screening criteria. Then, the eligible compounds were used for further analysis, and 893 putative targets were predicted in FSGD.

### 3.2 CRF Target Prediction and the Common Targets

**FIGURE 2:**
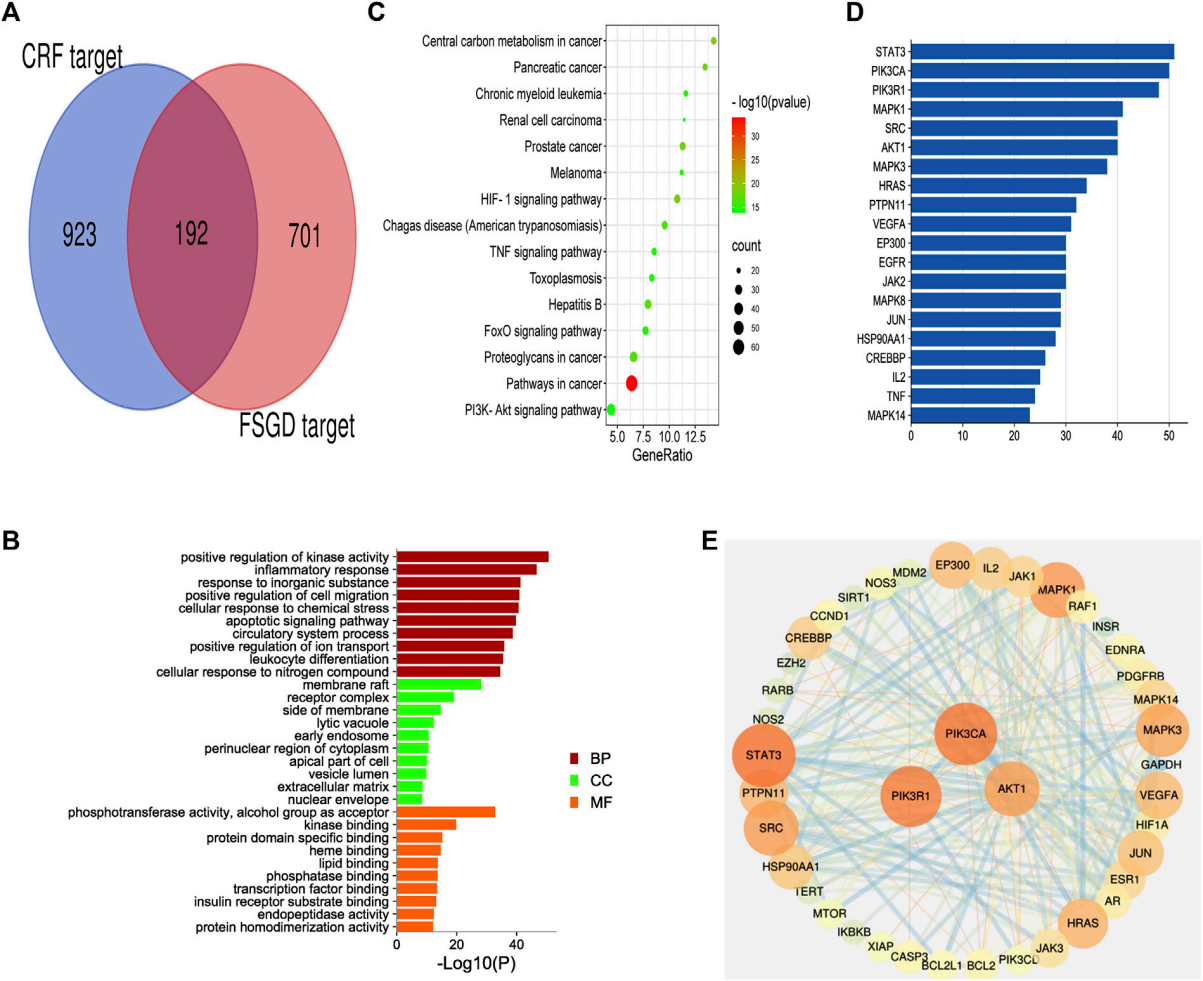
The first dimension-network pharmacology study of FSGD in CRF. **(A)** Venn diagram of common targets in FSGD and CRF. **(B)** GO enrichment analysis. *X*-axis represents the different values of −log10(p). *Y*-axis represents the name of biological processes. **(C)** The top 15 KEGG pathway enrichment dot-plot diagram. *X*-axis represents the ratio of enriched target genes/background genes. *Y*-axis represents the term of enriched pathways. The sizes of the dots indicate the number of target genes in a certain pathway, and the colors of the dots reflect the different values of −log10(p). **(D)** The construction of protein-protein interaction network. Edges represent protein-protein interactions, edge thickness indicates the strength of data support, the larger the node, the higher the degree value. These circles represent the targets that interact with AKT1. **(E)** The common targets-bar plot diagram. *X*-axis represents the number of nodes connected and *Y*-axis represents the target gene symbol.

A total of 8596 CRF targets were obtained from the genecards database. The higher relevance score indicated a better correlation between the gene and CRF. The 1,115 targets’ scores were more than two-fold of the average value, which was considered as the value of candidate therapeutic targets. After comparing the targets of FSGD with those of CRF, 192 matched targets were identified, such as EGFR, TNF and AKT1, and a venn diagram was plotted (Figure 2A). The first-dimension common targets were regarded as the potential therapeutic targets for FSGD against CRF.

### 3.3 GO and KEGG Enrichment Analysis

The common target genes were analyzed online using the bioinformatics software metascape, and the molecular function (MF), cell components (CC) and biological processes (BP) of these genes were analyzed by GO analysis (Figure 2B). After GO analysis, inflammatory response, apoptotic signaling pathway and extracellular matrix were found to be involved in the process of CRF (Taewon et al., 2016). The KEGG pathway enrichment analysis was performed by enhio (http://www.ehbio.com/) (*p* ≤ 0.05) (Figure 2C). Among the top 15 signaling pathways, PTEN/PI3K/AKT/NF-κB was involved in cell apoptosis and proliferation, inflammation, and fibrosis in the process of CRF (Song et al., 2020). Previous studies have shown that FSGD can improve renal function and inhibit renal fibrosis in CRF rats through the ACE-Ang II-AT1R axis in the RAS system, while RAS is closely related to the PI3K/AKT pathway in the liver (Molinaro et al., 2019; Xu J et al., 2021; Xu K et al., 2021). Based on the network pharmacological analysis and previous experimental studies, we speculate that the mechanism of FSGD in the treatment of CRF may be through the regulating of the PTEN/PI3K/AKT/NF-κB signaling pathway (Supplementary Figure S1).

### 3.4 The First-Dimension Network Visualization for the Theoretical Active Ingredients for FSGD in CRF

To further verify our conjecture, a string platform was employed to explore the interaction among the common 192 targets, and a PPI network was constructed by cytoscape3.7.2 (Supplementary Figure S2). This network comprised 168 nodes (ABCG2, ABCC1, PON1, CETP, PSEN1. GAA were concealed as disconnected nodes in the network) and 874 edges, with an average degree value of 10.40. The higher density connections representing the hub genes in network clusters of FSGD in CRF. STAT3, PIK3CA, PIK3R1, MAPK1, SRC and AKT1 were the top six target genes in the PPI network (Supplementary Table S2). PIK3CA, PIK3R1 and AKT1 were related to the PTEN/PI3K/AKT/NF-κB pathway, and AKT1 was a key gene, its targets are shown in Figure 2D, and the hunt hub genes are obtained in Figure 2E.

### 3.5 The Second-Dimension Network Pharmacological Analysis of Effective Compounds Obtained by UHPLC-MS/MS

**FIGURE 3:**
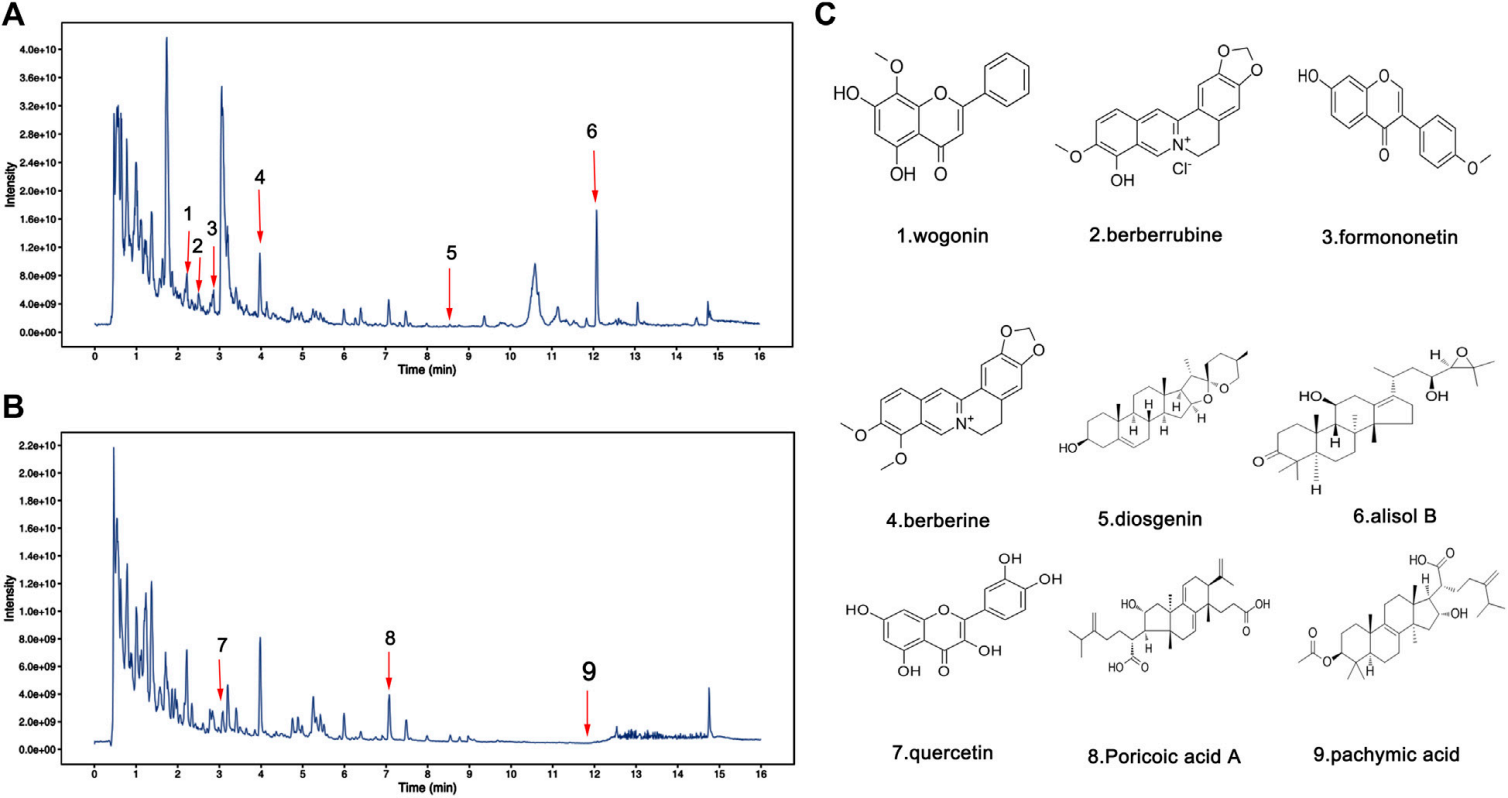
Identification of chemical components in water extract solutions of FSGD by ultra-high performance liquid chromatography tandem mass spectrometry (UHPLC–MS/MS). Total ion chromatography in positive **(A)** and negative **(B)** ion modes for FSGD samples as shown. The number corresponds to the compound on the left. **(C)** Molecular structure of constituents.

**FIGURE 4:**
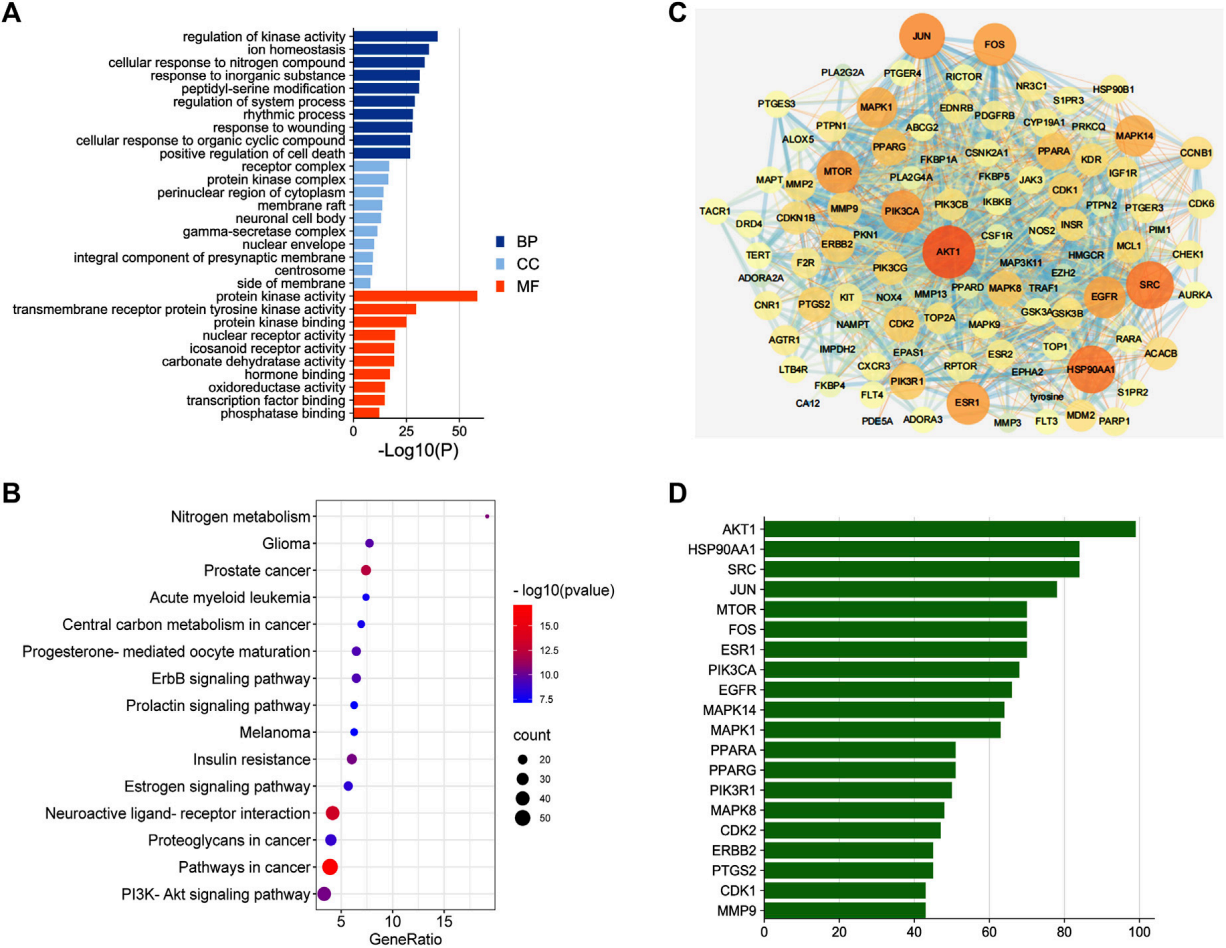
The second dimension-network pharmacology study of the detected ingredients of FSGD in CRF. **(A)** GO enrichment analysis. **(B)** The top 15 KEGG pathway enrichment dot-plot diagram. **(C)** The construction of protein-protein interaction network. **(D)** Common Targets-bar plot diagram.

To identify the major chemical components, the FSGD samples were analyzed using UHPLC-MS/MS. The total positive (Figure 3A) and negative (Figure 3B) ion chromatograms of FSGD demonstrated the chemical composition of all compounds, and we found that 40 compounds were found in FSGD (Supplementary Table S3). Nine candidate compounds were screened using the previous screening criteria, and their targets were predicted (Figure 3C), and the network pharmacology analysis was then carried out. PPI results show that AKT1was the top target gene and the central gene of the network. In addition, the results of GO, KEGG, and PPI all involved in the pathway of PTEN/PI3K/AKT/NF-κB (Figures 4A–D).

### 3.6 FSGD Protects Renal Function

**FIGURE 5:**
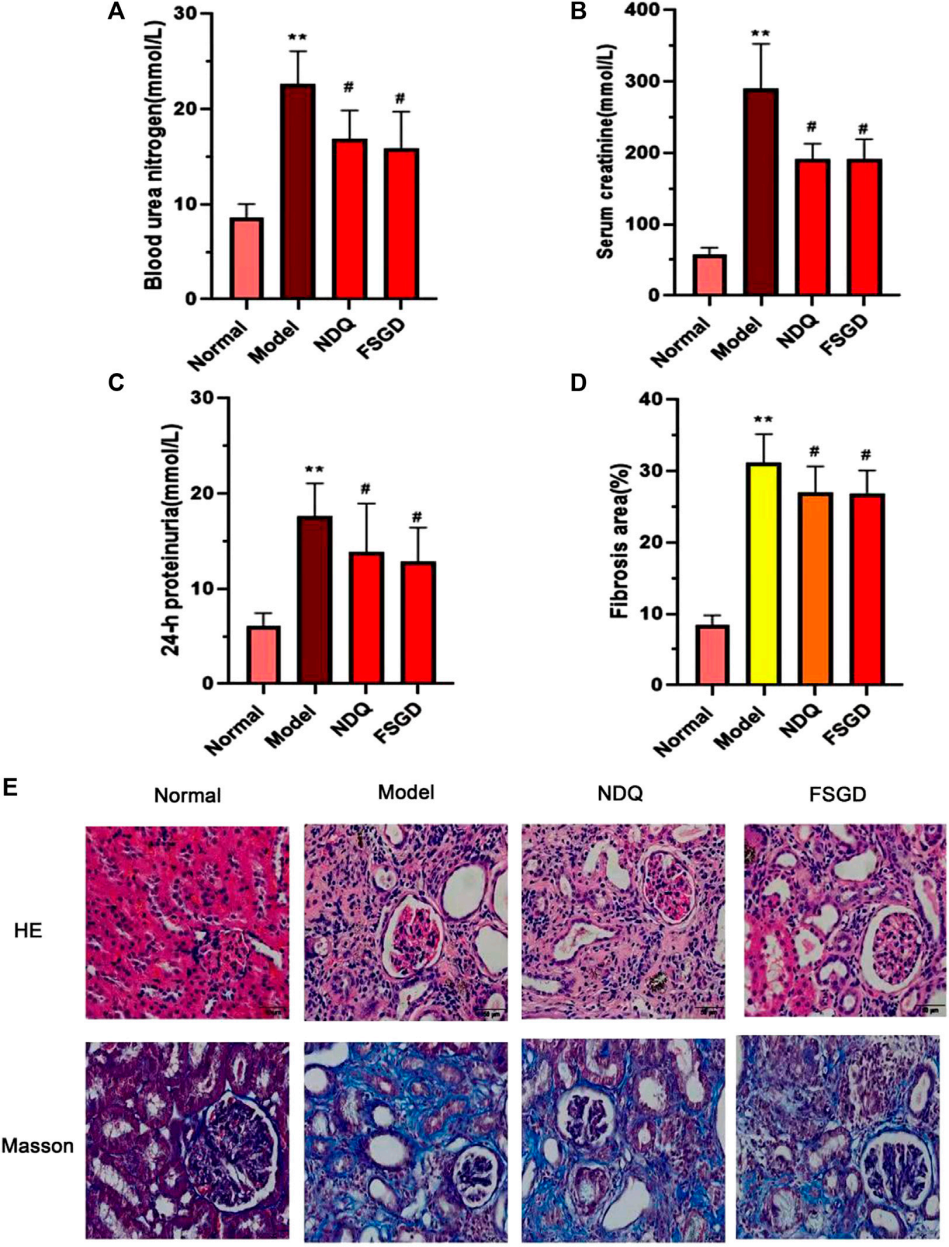
Effects of FSGD on renal pathological injury and renal fibrosis. **(A–C)** Effects of FSGD on serum concentrations of BUN CR and 24-h urinary protein. **(D)** Renal pathological changes were analyzed by hematoxylin-eosin and Masson staining was used to analyze collagen fibers in the kidney (×400). **(E)** The collagen fibers deposition was statistically analyzed with ImageJ software. The data are expressed as: compared with the normal group; Data were presented as the means ± SD. *p < 0.05, **p < 0.01; versus normal group; ^##^ *p* < 0.01, ^#^ *p* < 0.05, compared with the model group, respectively.

Change in serum biochemical parameters are related to kidney toxicity parameters in CRF (Firouzi and Haghighatdoost, 2018). Renal failure causes a decline in glomerular filtration function and further lead to the increase of the serum Cr and Bun (Cai et al., 2018). The 24-h urinary protein quantification, serum Cr, and BUN levels were used to examine the kidney function. Compared with the control, the 24-h urine protein quantification, serum Cr, and BUN of the model rats were significantly increased (*p* < 0.01), which indicated that the CRF rat model was established successfully in our experiment. However, FSGD and NDQ significantly reduced these CRF indicators (*p* < 0.05), and the improvement in the FSGD group was obvious (Figures 5A–C). These results show that FSGD can improve CRF symptoms and renal function, which is in line with previous research (Yang et al., 2017; Xu J et al., 2021; Xu K et al., 2021; Tong et al., 2020).

### 3.7 FSGD Alleviates Renal Pathological Injury and Renal Fibrosis

In this study, we evaluated the pathological changes in the kidney from different perspectives by HE (Figure 5D). In contrast to the normal group, the pathological morphology of the kidney in the model group was significantly changed, the renal tubules were enlarged or atrophied, many inflammatory cells were infiltrated, and the lumen was blocked by adenine crystal deposition. Meanwhile, NDQ and FSGD alleviate the above pathological injuries. In masson staining, red color indicates muscle fibers and blue indicates collagen fibers. Compared with the control, a significant accumulation of collagen fibers was found in the kidney tissue of the model group (*p* < 0.01); instead, FSGD significantly improved renal fibrosis (*p* < 0.05, Figure 5E).

### 3.8 Effects of FSGD on PI3K/AKT Pathways in CRF Model

**FIGURE 6:**
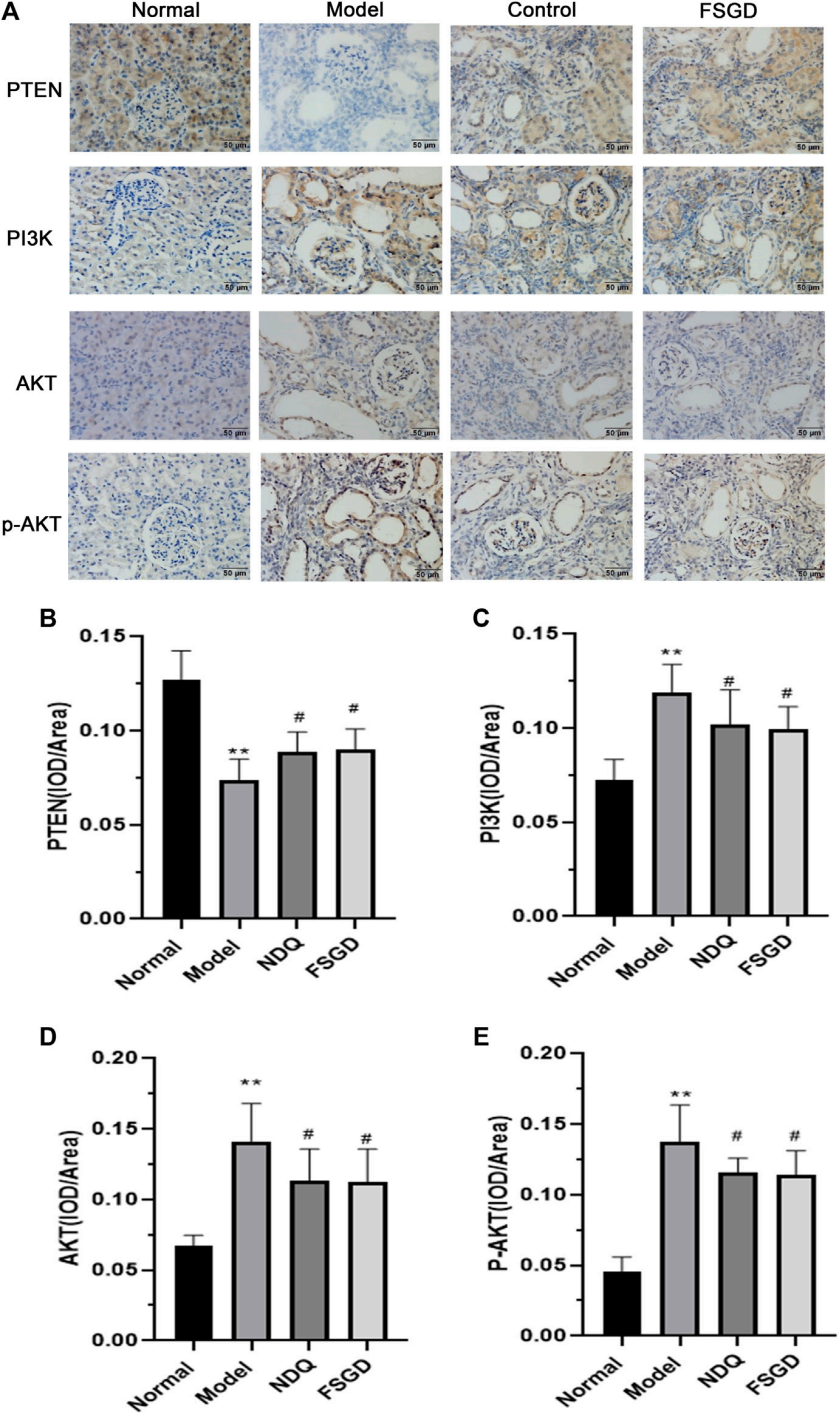
Effects of FSGD on PI3K/AKT pathway by immunohistochemistry. **(A)** The immunohistochemistry staining of PI3K/AKT pathway. **(B–E)** Image-Pro Plus software was used to statistically analyze the immunohistochemical staining results of PTEN, PI3K, AKT and p-AKT, respectively. The data are expressed as: compared with the normal group; *p < 0.05, **p < 0.01; versus normal group; ^##^ *p* < 0.01, ^#^ *p* < 0.05, compared with the model group, respectively.

The results showed that the expression of PTEN was significantly decreased in the model group by immunohistochemical determination (*p* < 0.01), but FSGD significantly increased the expression of PTEN (*p* < 0.05, Figures 6A,B), and which indicated that the PTEN is mainly regulated by the PI3K/AKT pathways. After immunohistochemistry analysis, the expression of PI3K, AKT, and p-AKT in the kidney are both significantly increased in model group (*p* < 0.01), FSGD and NDQ significantly upregulated the expression of PTEN and reduced the expression of PI3K, AKT, and p-AKT by inhibiting the PI3K/AKT pathway (*p* < 0.05, Figures 6C–E). Zheng et al. have shown that L-Carnitine can eliminate tacrolimus-induced PI3K/AKT activation and PTEN inhibition and counteract oxidative stress-mediated programmed cell death (Zheng et al., 2021). So, L-Carnitine could reduce the renal injury caused by TAC, our research consistent with the previous studies (Zheng et al., 2021).

**FIGURE 7:**
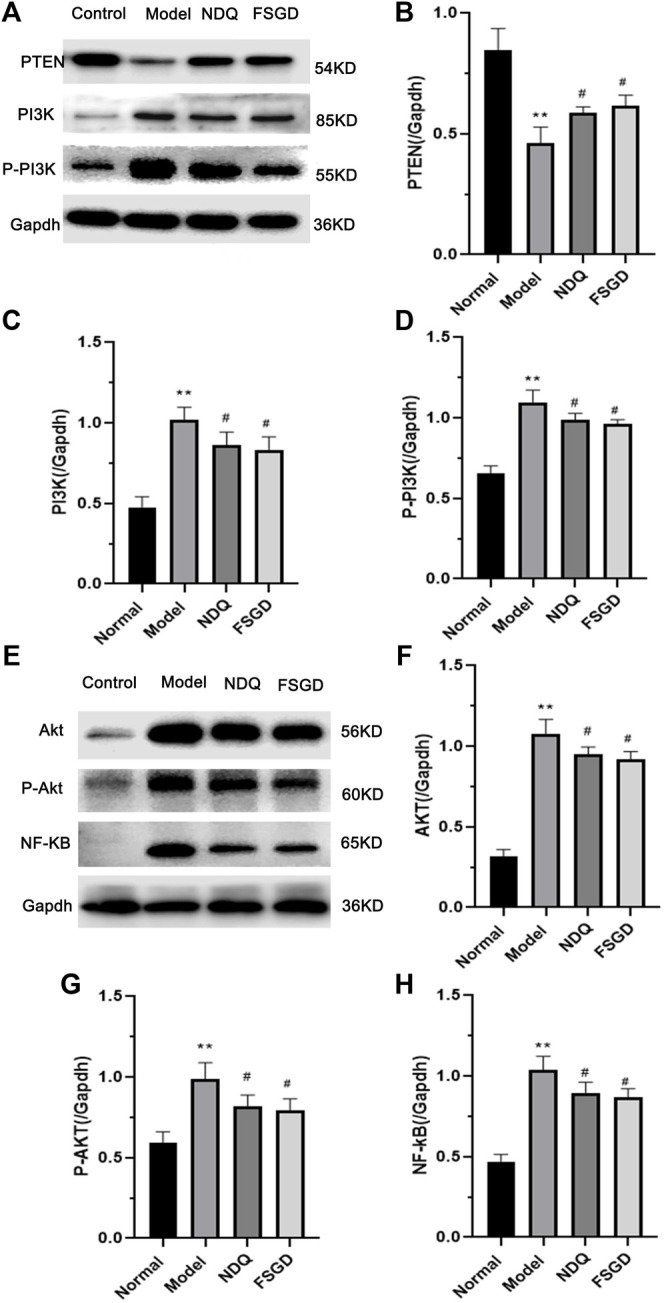
The protein analysis of Pi3k/Akt pathway for FSGD in CRF. **(A,E)** Western blotting of PTEN, PI3K, p-PI3K, AKT, p-AKT and NF-κB in the kidney. [**(B–D)** and **(F–H)**] The relative expression concentration analysis for PTEN, PI3K, p-PI3K, AKT, p-AKT and NF-κB in the kidney. Compared with the normal group, *p < 0.05, **p < 0.01; versus normal group; ^##^ *p* < 0.01, ^#^ *p* < 0.05, compared with the model group, respectively.

**FIGURE 8:**
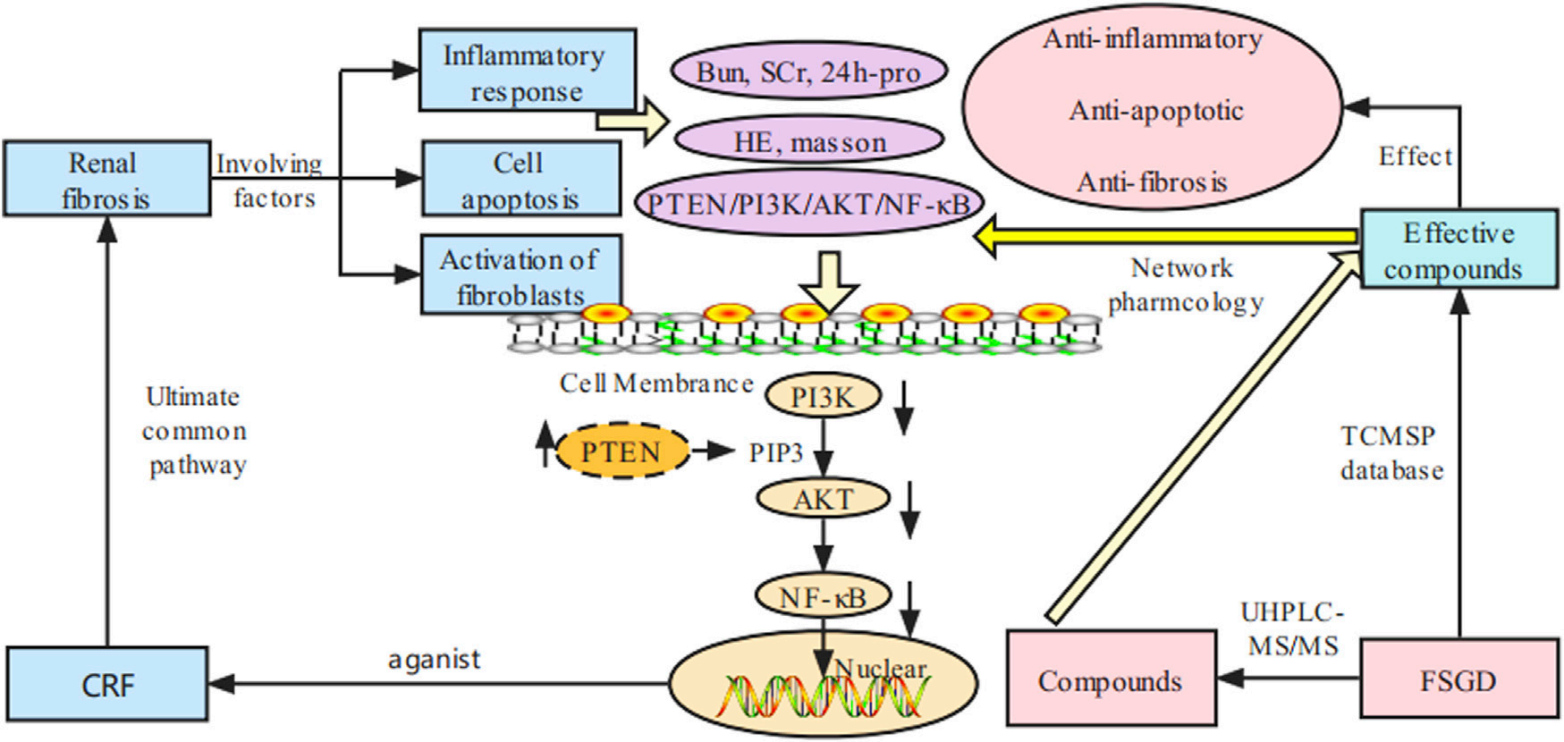
Schematic representation of molecular pathway of FSGD in CRF.

To confirm our hypothesis, the key factor PTEN and its upstream factors PI3K and p-PI3K in the kidney were detected using western blotting. The expression of PTEN was significantly decreased in the model group (*p* < 0.01), but it significantly increased after FSGD and NDQ treatment (*p* < 0.05, Figures 7A–D), compared with the modern group. The expressions of PI3K and p-PI3K were significantly increased in the model group, (*p* < 0.01), but significantly decreased after FSGD and NDQ treatment (*p* < 0.05, Figures 8A–D), compared with the model group. These similar results can be found in previous study (Haitao et al., 2021; Huo et al., 2021).

To further explain the mechanism of FSGD on the PTEN/PI3K/AKT/NF-κB pathway, the downstream factors AKT, p-AKT, and NF-κB were measured because these factors play an important role in renal fibrosis. Compared with the normal group, AKT, p-AKT, and NF-κB were significantly increased in the model group (*p* < 0.01), indicating that the PTEN/PI3K/AKT/NF-κB pathway was activated in the kidneys of CRF rats, while the expressions of AKT, p-AKT, and NF-κB were significantly decreased after FSGD and NDQ treatment (*p* < 0.05, Figures 8E–H). The result of our study is the same as the earlier conclusion (Ba et al., 2020).

## 4 Discussion

TCM therapy has become increasing popular in China and other Asian countries for refractory diseases (Peng et al., 2017; Pan et al., 2019), and network pharmacology is a new strategy to explore the mechanism of drug treatment of diseases (Li et al., 2020). In our study, the candidate FSGD compounds and targets against CRF were predicted using network pharmacology, which yielded 121 effective compounds and 192 targets. However, this has only based on the database analysis and has not been verified by experiments. Therefore, we verified the authenticity and feasibility of two-dimensional network drug prediction through experiments. An overview of this study is shown in Figure 8.

The results indicated that the main FSGD compounds involved in the treatment of CRF were quercetin, formononetin, and poria acid. The previous study found that quercetin could reduce epithelial to mesenchymal transition (EMT), extracellular matrix (ECM) deposition, and cell proliferation to exert anti-fibrosis effects in NRK-52E cells treated with TGF- β1 and in renal obstruction rats through the overactive Hedgehog pathway (Liu et al., 2019). Formononetin and pachymic acid have anti-inflammatory and antioxidant effects (Feng et al., 2020; Oza and Kulkarni, 2020). In a word, these compounds have been shown to exert anti-inflammatory, anti-fibrosis, and antioxidant effects in CRF treatment, which could play a positive role (Ullah and Basile, 2019). And these selected compounds in this study can lay the foundation for the development of new drugs to treat CRF in the future. This study, like a beacon before dawn, points out the direction for our follow-up research to treat CRF as soon as possible.

Further analysis showed that AKT1, EGFR, and TNF may be potential targets for FSGD in the treatment of CRF, and the PPI network indicated that AKT1 was one of the central genes related to 40 highly valuable targets and is closely related to fibrosis, and it may be a new target for CRF (Lin et al., 2020). AKT1 can promote EGFR ubiquitination and degradation to prevent renal intertubule fibrosis (Zhu et al., 2020). Neutralizing inflammatory cytokines (TNF) can reduce the level of FGF23 in animal models of chronic kidney disease (CKD) and high levels of FGF23 are associated with increased cardiovascular morbidity and mortality in patients with CKD (Egli-Spichtig et al., 2019). Therefore, TNF can be used as a potential therapeutic target for CRF, thereby reducing mortality in patients with CRF. Moreover, the KEGG results suggest that the PTEN/PI3K/AKT/NF-κB signal pathway may be a potential mechanism of FSGD in the treatment of CRF, and the inhibition of the PTEN/PI3K/AKT/NF-κB pathway can reduce inflammation, anti-apoptosis and reduce fibrosis in 60 diabetic C57BL/6 mice (Song et al., 2021). And then, we obtained the effective components of FSGD by mass spectrometry and carried out the second-dimension network pharmacological analysis, and the results were consistent with the results of theoretical network pharmacology analysis. Therefore, we speculate that PTEN/PI3K/AKT/NF-κB pathway is an important mechanism of FSGD in the treatment of CRF.

Renal fibrosis is the ultimate common pathway of progressive nephropathy, characterized by excessive accumulation and deposition of the renal interstitium and glomerular extracellular matrix, which ultimately leads to end-stage renal failure (Du et al., 2020). Renal fibrosis is a pathological process driven by multiple factors, including inflammatory response, immune cell apoptosis, proliferation, and activation of fibroblasts (Taewon et al., 2016). Evidence that PI3K/AKT/PTEN signaling pathway is involved in the pathogenesis of many renal diseases, and the PI3K/AKT/PTEN signal axis links oxidative stress with programmed cell death (Zheng et al., 2021). The inhibition of the over-activation of PI3K/AKT signaling pathway can protect the integrity of the podocyte skeleton and foot process in rats through low renal pathological injury and reduced urinary protein (Lin et al., 2019). And PTEN/PI3K/AKT pathway can also reduce the expression of collagen I (COL I), fibronectin (FN) and α-smooth muscle actin (α-SMA) mediated by fibrotic factor TGF-β1, reduce inflammation and oxidative stress in the kidney, and thereby inhibit renal interstitial fibrosis (Song et al., 2020). Furthermore, PTEN can regulate endogenous factor transforming growth factor TGF-β to reduce the accumulation of ECM (Tang et al., 2020) and EMT (Liu B et al., 2018). These findings show that PTEN is closely related to renal tissue fibrosis (Wang Y et al., 2020). In this study, two-dimensional network pharmacological analysis was used to predict that PTEN/PI3K/AKT/NF-κB is an important mechanism of FSGD in the treatment of CRF. David et al have shown that uremia can lead to significant disturbances in the AKT system. Some studies have shown that there are disturbances in total AKT and phosphorylated AKT in uremic rats. The consequences of AKT activation vary greatly depending on the activation pathway, the duration of activation and the specific subtypes affected. The results of acute and chronic activation of AKT pathway are different. Chronic AKT activation is harmful, and chronic overexpression of AKT1 in LVH can lead to cardiac fibrosis associated with AKT1 (Matsui et al., 2002; Nagoshi et al., 2005; Shioi et al., 2002; Li et al., 2007; Semple et al., 2011). It was found that the expression of total AKT and total PI3K protein increased in the kidneys of CRF rats, which may be related to the complex pathogenesis and pathway of PTEN in CRF, which is also consistent with the previous studies (Gao et al., 2021). In addition, PTEN can inhibit the downstream AKT and reduce the recruitment of AKT-mediated monocytes to injured kidney, as a negative regulator of PI3K (Haddadi et al., 2018; Sasaki et al., 2021). The lower expressions of PI3K/AKT inhibit the downstream NF-κB signal transduction, which could reduce the production of inflammatory factors and various chemokines in renal fiber formation and prevent the transformation of fibroblasts into myofibroblasts with stronger secretion and proliferation ability to improve kidney injury and reduce renal fibrosis and glomerular sclerosis, etc. (Liu X et al., 2018; He et al., 2019). Wang et al have founded that PI3K, p-PI3K, AKT, and p-AKT increased significantly in the rat model of ulcerative colitis with yang deficiency of spleen and kidney, while their express levels decreased significantly after drug intervention (Wang et al., 2021). And some researchers found that NF-κB is highly activated in inflammation (Amini-Farsani et al., 2021; Ilchovska and Barrow, 2021). And NF-κB is the downstream of the PTEN/PI3K/AKT pathway (Supplementary Figure S1). Compared with the normal group, the NF-κB protein expression in the model group was significantly increased. The expression of NF-κB protein was significantly reduced after drug intervention. Our results are consistent with the above reported (Wang et al., 2021). But the study of the PTEN/PI3K/AKT/NF-κB pathway was extremely rare in the CRF. Therefore, this study explored the mechanism of FSGD against renal fibrosis in rats with CRF.

In our study, to improve the predication accuracy of network pharmacology, the first-dimensional network pharmacology is based on the theoretical prediction of the mechanism of drug action on the disease, and the second-dimensional network pharmacology is based on the actual potential effective compounds in prescription on CRF for network analysis with the UHPLC-MS/MS identification technology. And then, the GO results of both network pharmacology show that it is closely related to protein kinase, and many studies have also shown that protein kinase plays an important role in CRF (Yang et al., 2019; Wada et al., 2020). At the same time, two-dimensional network pharmacology KEGG results show that PTEN/PI3K/AKT/NF-κB pathway plays an important role in CRF (Song et al., 2020). PPI results show that AKT is a key gene in protein interaction. Studies have shown that AKT is the key node of PTEN/PI3K/AKT/NF-κB signaling pathway (Xu J et al., 2021; Xu K. et al., 2021). In addition, inflammatory response, hormones, and apoptosis are involved in the process of CRF (Li H et al., 2021) (Wang et al., 2019; Yazgan et al., 2021). HIF-1 signaling pathway, ErbB signaling pathway, estrogen signaling pathway and other pathways need to be further studied to find new targets for the treatment of CRF. Two-dimensional network pharmacology combines theory with practice to successfully predict the important mechanism of FSGD acting on CRF, which lays a solid foundation for follow-up molecular experiments. Recently, many literatures have also shown that many new drugs and new targets for the treatment of diseases have been developed based on network pharmacology (Kibble et al., 2015; Li R et al., 2021). Based on the two-dimensional network pharmacology, we also found that there are many components and targets to treat CRF. These findings provide a new direction for the drug development. In this study, we conducted further dual-dimensional network pharmacology analysis on the possible mechanism of diseases and prescription of Chinese medicine and found that the PI3K/AKT pathway is very important for FSGD to treat CRF. Combined with animal experiments, we found that FSGD significantly enhanced renal function and improved renal fibrosis to produce renal function recovery and renal tissue repair. Immunohistochemical and western blot analyses showed that FSGD can regulate the PTEN/PI3K/AKT/NF-κB signaling pathway through increasing PTEN expression and inhibiting the activation of PTEN/PI3K/AKT/NF-κB, compared with the model rats. Moreover, the results showed that the treatment effect of FSGD formula was better than that of the positive drug NDQ.

In conclusion, this work systematically identified the compounds and studied the mechanisms of FSGD in the treatment of CRF using dual-dimensional network pharmacology and *in vivo* experiments. Our strategy found that the effect of FSGD on reducing renal fibrosis and renal injury in CRF rats may be related to the inhibition of the PTEN/PI3K/AKT/NF-κB signaling pathway. Two-dimensional network pharmacology is a new strategy for exploring the mechanism of complex diseases and drug multi-targets, which can help us to find the most probable mechanism of drug treatment more accurately and provide a more sufficient theoretical basis for further experiments. More experiments are needed to provide more evidence in the future.

## Data Availability

The original contributions presented in the study are included in the article/Supplementary Material, further inquiries can be directed to the corresponding authors.

## Abbreviations

BUN, urea nitrogen; Cr, creatinine; CRF, chronic renal failure; DL, drug-likeness; FSGD, Fushengong decoction; NDQ, Niaoduqing; OB, oral bioavailability; TCM, traditional Chinese medicine; TCMSP, traditional Chinese medicine systems pharmacology.
