# Supplementary material for: Effect of Fushengong Decoction on PTEN/PI3K/AKT/NF-κB Pathway in Rats With Chronic Renal Failure via Dual-Dimension Network Pharmacology Strategy
Source: Front Pharmacol. 2022 Mar 15;13:807651. doi: 10.3389/fphar.2022.807651 (PMC8965284; doi:10.3389/fphar.2022.807651)
Supplement: Supplementary file 1 [file DataSheet1.docx]

**Supplementary Materials**


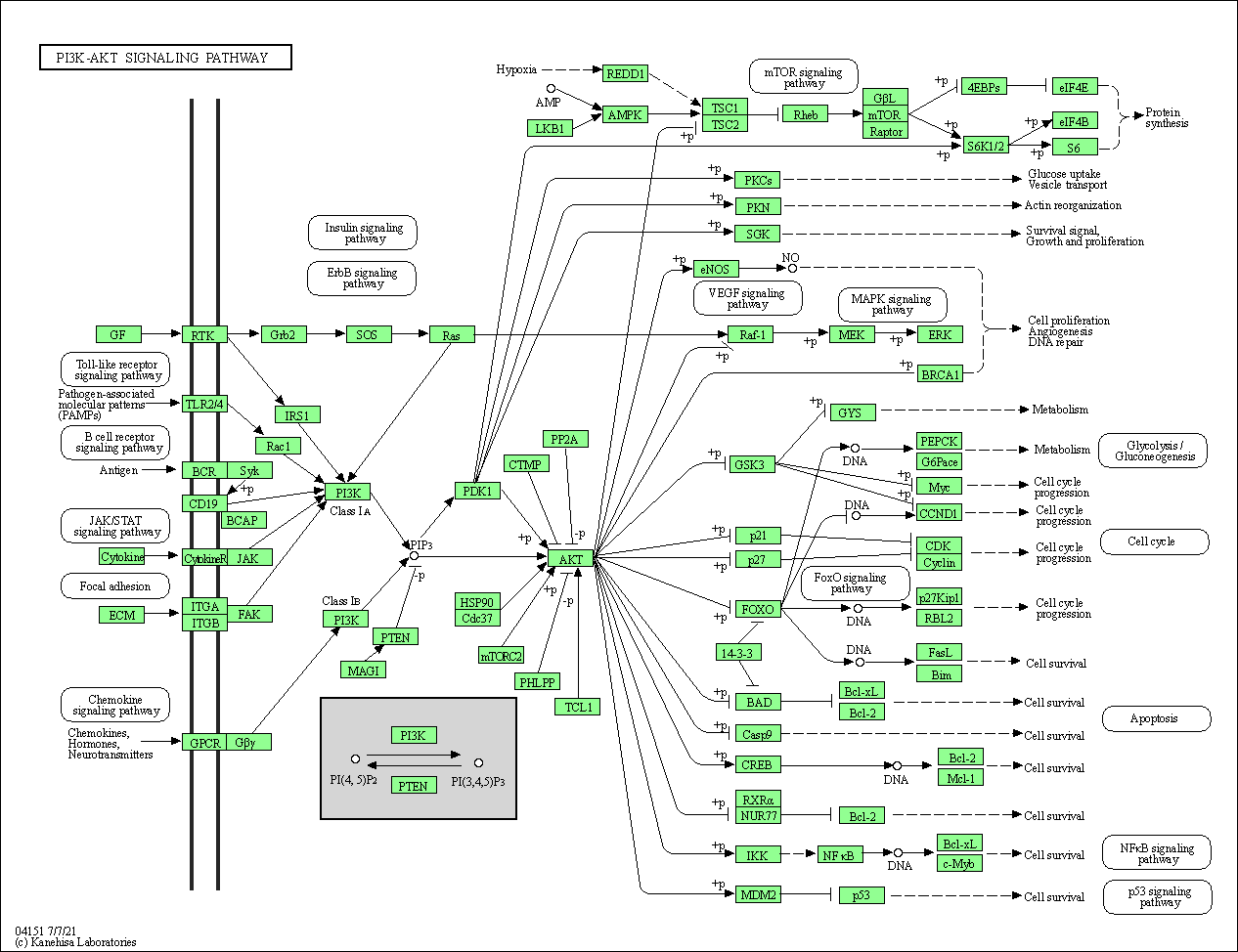


**Figure S1.** The pathway of PI3K/AKT.


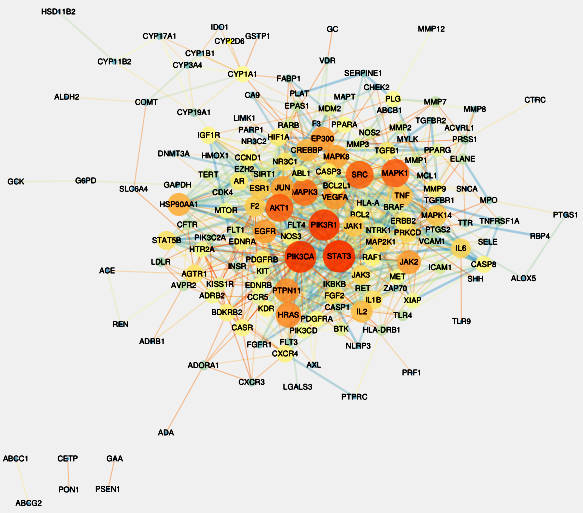


**Figure S2.** The construction of protein-protein interaction network. Edges represent protein-protein interactions, edge thickness indicates the strength of data support, the larger the node, the higher the degree value.

**Table S1.** The Chinese herbal medicines in Fushengong decoction (FSGD).

| Pinyin name | Scientific name | Batch number | Composition (%) |
| --- | --- | --- | --- |
| Huangqi (HQ) | *[Astragalus mongholicus](https://mpns.science.kew.org/mpns-portal/plantDetail?plantId=661222&query=Radix+Astragali&filter=&fuzzy=false&nameType=all&dbs=wcsCmp)* [Bunge](https://mpns.science.kew.org/mpns-portal/plantDetail?plantId=661222&query=Radix+Astragali&filter=&fuzzy=false&nameType=all&dbs=wcsCmp) | 200409 | 21.74 |
| Dihuang (DH) | *[Rehmannia glutinosa](https://mpns.science.kew.org/mpns-portal/plantDetail?plantId=527243&query=dihuang&filter=&fuzzy=false&nameType=all&dbs=wcsCmp)* [(Gaertn.) DC.](https://mpns.science.kew.org/mpns-portal/plantDetail?plantId=527243&query=dihuang&filter=&fuzzy=false&nameType=all&dbs=wcsCmp) | 200217 | 6.52 |
| Shanyao (SY) | *[Dioscorea oppositifolia](https://mpns.science.kew.org/mpns-portal/plantDetail?plantId=240599&query=Shanyao&filter=&fuzzy=false&nameType=all&dbs=wcs)* [L.](https://mpns.science.kew.org/mpns-portal/plantDetail?plantId=240599&query=Shanyao&filter=&fuzzy=false&nameType=all&dbs=wcs) | 200612 | 8.70 |
| Shanzhuyu (SZY) | *Cornus officinalis* Siebold & Zucc. | 200318 | 6.52 |
| Cheqian (CQ) | *[Plantago asiatica](https://mpns.science.kew.org/mpns-portal/plantDetail?plantId=571414&query=Cheqian&filter=&fuzzy=false&nameType=all&dbs=wcsCmp)* [L.](https://mpns.science.kew.org/mpns-portal/plantDetail?plantId=571414&query=Cheqian&filter=&fuzzy=false&nameType=all&dbs=wcsCmp) | 200609 | 6.52 |
| Fuling (FL) | *Poria Cocos (Schw.)* Wolf | 202001003 | 8.70 |
| Cangzhu (CZ) | *[Atractylodes lancea](https://mpns.science.kew.org/mpns-portal/plantDetail?plantId=872479&query=Baizhu&filter=&fuzzy=false&nameType=all&dbs=wcsCmp)* [(Thunb.) DC.](https://mpns.science.kew.org/mpns-portal/plantDetail?plantId=872479&query=Baizhu&filter=&fuzzy=false&nameType=all&dbs=wcsCmp) | 191121 | 6.52 |
| Niuxi (NX) | *[Achyranthes bidentata](https://mpns.science.kew.org/mpns-portal/plantDetail?plantId=617516&query=Radix+Achyranthis+Bidentatae&filter=&fuzzy=false&nameType=all&dbs=wcsCmp)* [Blume](https://mpns.science.kew.org/mpns-portal/plantDetail?plantId=617516&query=Radix+Achyranthis+Bidentatae&filter=&fuzzy=false&nameType=all&dbs=wcsCmp) | 200702 | 6.52 |
| Duzhong (DZ) | *Eucommia ulmoides* Oliv. | 202006001 | 6.52 |
| Mudanpi (MDP) | *[Paeonia × suffruticosa](https://mpns.science.kew.org/mpns-portal/plantDetail?plantId=519321&query=Cortex+Moutan+Radicis&filter=&fuzzy=false&nameType=all&dbs=wcs)* [Andrews](https://mpns.science.kew.org/mpns-portal/plantDetail?plantId=519321&query=Cortex+Moutan+Radicis&filter=&fuzzy=false&nameType=all&dbs=wcs) | 200103 | 6.52 |
| Shuizhi (SZ) | *Hirudo* Whitman | 191218 | 4.35 |
| Zexie (ZX) | *Alisma plantago-aquatica subsp. orientale* (Sam.) Sam. | 191227 | 4.35 |
| Huangbai (HB) | *[Phellodendron chinense](https://mpns.science.kew.org/mpns-portal/plantDetail?plantId=541751&query=Huangbai&filter=&fuzzy=false&nameType=all&dbs=wcsCmp)* [C.K. Schneid.](https://mpns.science.kew.org/mpns-portal/plantDetail?plantId=541751&query=Huangbai&filter=&fuzzy=false&nameType=all&dbs=wcsCmp) | 200706 | 6.52 |

**Table S2.** Parameters of the top 20 genes in the PPI network.

| Number | Gene | Betweenness centrality | Closeness centrality | Degree |
| --- | --- | --- | --- | --- |
| 1 | STAT3 | 0.12 | 0.53 | 51 |
| 2 | MAPK1 | 0.09 | 0.51 | 41 |
| 3 | PIK3CA | 0.08 | 0.50 | 50 |
| 4 | PIK3R1 | 0.07 | 0.50 | 48 |
| 5 | MAPK3 | 0.04 | 0.50 | 38 |
| 6 | AKT1 | 0.07 | 0.50 | 40 |
| 7 | SRC | 0.06 | 0.49 | 40 |
| 8 | VEGFA | 0.06 | 0.48 | 31 |
| 9 | HSP90AA1 | 0.04 | 0.48 | 28 |
| 10 | JUN | 0.04 | 0.47 | 29 |
| 11 | EGFR | 0.06 | 0.47 | 30 |
| 12 | ESR1 | 0.06 | 0.46 | 22 |
| 13 | PTPN11 | 0.02 | 0.46 | 32 |
| 14 | MAPK8 | 0.02 | 0.46 | 29 |
| 15 | EP300 | 0.04 | 0.45 | 30 |
| 16 | HRAS | 0.03 | 0.45 | 34 |
| 17 | MAPK14 | 0.02 | 0.45 | 23 |
| 18 | CREBBP | 0.08 | 0.45 | 26 |
| 19 | JAK2 | 0.02 | 0.45 | 30 |
| 20 | IL2 | 0.02 | 0.44 | 20 |

**Table S3**. Compounds from UHPLC-MS/MS in FSGD

| Mol ID | Molecule name | Origin | Mzmed |
| --- | --- | --- | --- |
| MOL000043 | Atractylenolide i | BZ | 231.14 |
| MOL000045 | Atractylenolide iii | BZ | 247.13 |
| MOL004557 | Geniposide | CQ, NX, DZ | 433.14 |
| MOL003690 | Ajugol | DH | 393.14 |
| MOL003717 | Methyl palmitoleate | DH | 267.23 |
| MOL000843 | stachyose | DH, ZX, | 665.21 |
| MOL000842 | sucrose | DH, ZX, | 341.11 |
| MOL001500 | LOLIOLIDE | DZ | 197.12 |
| MOL001668 | Geniposidic acid | DZ | 373.11 |
| MOL002049 | Ferulaldehyde | DZ | 179.07 |
| MOL000481 | Genistein | DZ | 269.05 |
| MOL000771 | p-coumaric acid | DZ | 165.05 |
| MOL006356 | Sorbitol | DZ | 181.07 |
| **MOL000289** | **Pachymic acid** | **FL** | 527.38 |
| **MOL000290** | **Poricoic acid A** | **FL** | 497.33 |
| MOL013352 | Obacunone | HB | 455.21 |
| MOL002665 | Ferulic Acid | HB | 193.05 |
| **MOL002894** | **Berberrubine** | **HB** | 322.11 |
| MOL003959 | Limonin | HB | 515.19 |
| MOL002642 | Phellodendrine | HB | 342.17 |
| MOL000390 | Daidzein | HQ | 255.07 |
| MOL000391 | Ononin | HQ | 431.13 |
| **MOL000392** | **Formononetin** | **HQ** | 291.06 |
| MOL000441 | LUPENONE | HQ | 425.38 |
| **MOL000098** | **Quercetin** | **HQ** | 301.04 |
| MOL000394 | Choline | HQ, SY, ZX | 104.11 |
| MOL007006 | oxypaeoniflorin | MDP | 495.15 |
| MOL003867 | Paeonolide | MDP | 459.15 |
| MOL000675 | Oleic acid | MDP, HB | 281.25 |
| **MOL001454** | **Berberine** | **NX** | 336.12 |
| **MOL000173** | **Wogonin** | **NX** | 283.06 |
| MOL005325 | Ginsenoside Ro | NX | 955.49 |
| MOL000785 | Palmatine | NX | 352.15 |
| **MOL000546** | **Diosgenin** | **SY** | 415.32 |
| MOL001403 | Erucamide | SZY | 338.34 |
| MOL001680 | Loganin | SZY | 435.15 |
| MOL000635 | Vanillin | SZY, HB | 153.05 |
| MOL000223 | Caffeic acid | SZY, MDP, DZ, | 179.03 |
| MOL000828 | Alisol A | ZX | 535.36 |
| **MOL000830** | **Alisol B** | **ZX** | 495.35 |

Note: The blue color represent that these compounds are effective compounds.
